# Increases in Hepatitis C Virus (HCV) Treatment and Cure Among People Who Inject Drugs in the Seattle Area Between 2018 and 2022

**DOI:** 10.1007/s10461-026-05110-9

**Published:** 2026-04-01

**Authors:** Jenni L. Ebersberger, Judith I. Tsui, Maria A. Corcorran, Sara N. Glick

**Affiliations:** 1 University of Washington School of Medicine, Seattle, WA USA; 2 Division of General Internal Medicine, Department of Medicine, University of Washington, Seattle, WA USA; 3 Division of Allergy and Infectious Diseases, Department of Medicine, University of Washington, Seattle, WA USA; 4 Public Health – Seattle & King County, HIV/STI/HCV Program, Seattle, WA USA

**Keywords:** Hepatitis C virus (HCV), People who inject drugs, Continuum of care, HCV testing, HCV treatment

## Abstract

This study aimed to describe the current hepatitis C virus (HCV) continuum of care and characterize the prevalence of HCV among Seattle-area people who inject drugs (PWID), including an assessment of changes between 2018 and 2022. Cross-sectional data from the 2018 and 2022 Seattle-area National HIV Behavioral Surveillance (NHBS) surveys of PWID was included in this study. All participants completed a biobehavioral survey and were offered rapid HIV and HCV antibody testing. Among those who screened HCV antibody-positive, we estimated proportions for steps along the HCV care continuum, including proportions of PWID who had previously been told they had HCV, were treated, and cured. The 2018 and 2022 NHBS-PWID surveys included 533 and 495 participants, respectively. In 2022, 61% (303/495) of PWID tested HCV antibody-positive, compared with 71% (376/533) in 2018. Among those who were HCV antibody-positive, prior HCV testing was common in both years [94% (95% CI: 91%-96%) in 2018; 92% (95% CI: 89%-95%) in 2022]. The proportion reporting prior HCV diagnosis increased from 68% (95% CI: 63%-72%) in 2018 to 71% (95% CI: 66%-76%) in 2022. Among those diagnosed, treatment rose from 26% (95% CI: 20%-31%) in 2018 to 47% (95% CI: 40%-54%) in 2022, and cure increased from 18% (95% CI: 14%-23%) in 2018 to 33% (95% CI: 27%-40%) in 2022. Compared to 2018, in 2022 the proportion of PWID with HCV in the Seattle area who had been treated and cured nearly doubled. Sustained investment in HCV diagnosis and treatment is needed to strengthen the HCV continuum of care.

## Introduction

Hepatitis C virus (HCV) is a leading cause of morbidity and mortality globally, affecting an estimated 50 million people worldwide, with about one million new infections occurring per year [1]. Data from the 2009–2018 National Health and Nutrition Examination Survey (NHANES) indicated that the prevalence of current HCV infection (HCV RNA detected) among the general population in the U.S. is 1% [2]. However, this estimate is likely under-representative of the true HCV prevalence due to NHANES’ exclusion of some populations (e.g., persons living homeless, incarcerated populations) known to have high HCV prevalence [3]. In 2019, researchers estimated that approximately 4.1 million persons in the U.S. were HCV antibody-positive [3]. This prevalence of HCV is despite significant advances in HCV treatment regimens [4]. With the advent of all oral direct-acting antiviral (DAA) therapies in 2013, cure rates of over 95% can now be reached even among people who use drugs if they have access to and initiate these therapies [4].

Injection drug use (IDU) is the most common risk factor for the transmission of HCV due to transmission risk from sharing injection equipment [5, 6]. It has been estimated that 52% of people who inject drugs (PWID) globally are HCV antibody-positive and 39% are viremic [7]. The estimated 5.6 million PWID with chronic HCV infection globally have been identified as a vulnerable and difficult population to test, treat, and cure [7]. Steps to expand HCV treatment uptake among PWID are critical due to the potential adverse consequences of untreated HCV. Most (~ 60–80%) people who are acutely infected with HCV will not clear the virus and go on to develop chronic HCV infection [8]. If left untreated, chronic HCV infection can lead to cirrhosis, end-stage liver disease and hepatocellular carcinoma, among other complications [8]. Early HCV testing and treatment can reduce the spread of the virus and prevent these highly morbid complications [1].

Access to and coverage of DAAs is improving across the U.S.; however, gaps still exist in reaching and providing care to PWID [4]. Using data from the 2015 and 2018 Seattle-area National HIV Behavioral Surveillance (NHBS) system, we previously published two of the few U.S.-based analyses of the HCV continuum of care among PWID in the DAA era [9, 10]. In both studies, we found a high prevalence of prior testing among HCV-positive PWID in Seattle and King County, but large gaps in the proportion of people with HCV who had been treated and cured.

Since the previous data collection in 2018, there have been many impacts to the landscape of HCV care including the COVID-19 pandemic, simplified HCV treatment guidelines, and the emergence of “low-barrier” models of HCV treatment [11]. The pandemic introduced unique barriers to healthcare access; patients with HCV were far less likely to complete treatment, obtain laboratory tests, or achieve HCV cure [12]. Beyond changes and challenges through the COVID-19 pandemic, Washington State continues to work towards meeting its statewide plan for HCV elimination by 2030 through the “Hep C Free Washington” initiative launched in 2019. This plan reduced DAA prior authorization requirements for Washington Medicaid patients, intensified HCV surveillance databases, enhanced public health outreach, removed specialty consultation requirements, and secured a pricing agreement with AbbVie, the manufacturer of glecaprevir/pibrentasvir [13]. Similarly, global targets set by the World Health Organization (WHO) remain on the horizon, which aim to treat 80% of those infected with HCV and reduce new HCV infections by 90% and HCV related deaths by 65% between 2016 and 2030 [7]. With the many recent changes to HCV treatment landscape and an ongoing attempt to meet WHO and Washington State goals, an updated HCV continuum of care analysis is warranted.

This study aims to characterize the HCV prevalence among PWID in the Seattle area and update the HCV continuum of care in a dynamic, multi-faceted HCV landscape. Studying the HCV continuum of care aids in our understanding of gaps in reaching populations disproportionately affected by HCV, testing and diagnostic methods, treatment, and cure of HCV. For this study, we compared NHBS data from 2018 to the most recent 2022 data and evaluated differences in the continuum of care.

## Methods

### Study Sample

This study analyzed data from the 2022 Seattle-area NHBS survey, which focused on PWID, and compared estimates to previously reported findings from 2018 [10]. NHBS is a comprehensive system for biobehavioral surveillance in 20 metropolitan populations disproportionately affected by HIV and is conducted in rotating, annual cycles by the Centers for Disease Control and Prevention (CDC); these 3 annual cycles are considered a round. Venue-based, time-space sampling is used for the gay, bisexual, and other men who have sex with men (MSM) population. Respondent-driven sampling (RDS) is used for PWID and heterosexually active persons at increased risk for HIV infection (HET) populations. A standardized, anonymous questionnaire collects information on HIV-related behavioral risk factors (e.g., sexual behaviors, drug use), HIV testing, and use of prevention services (e.g., HIV pre-exposure prophylaxis). In each cycle, approximately 500 eligible persons from each participating area are interviewed and offered anonymous HIV testing [14]. The overview and methodology of NHBS has previously been published [14].

The 2018 and 2022 NHBS-PWID survey included people who were 18 years or older, reported injecting drugs in the past year, lived in King or Snohomish County WA, and were able to complete the survey in English or Spanish. Participants were recruited via RDS, a form of snowball sampling where participants are monetarily incentivized to recruit their peers. RDS for this study began with initial recruits, or “seeds,” identified by the study team and other persons who work with the target population through outreach. Seeds completed the NHBS survey and HIV/HCV testing and were then asked to recruit others using recruitment coupons. Participants were provided up to 5 coupons and received a brief training on who to recruit.

### Data Collection and HIV/HCV Testing

Trained interviewers performed data collection from July through December 2022 at two community field sites, one in King County and one in Snohomish County, WA. Eligible participants completed an informed consent process. Participants who consented completed an interviewer-administered survey, which included questions about sociodemographic characteristics, medical history, and sexual and drug-use practices. The NHBS survey is divided into a core and local survey. The core survey includes questions provided by the CDC, while the local survey allows each NHBS project area to collect data on their unique community contexts like specific local risk factors or additional health outcomes like HCV. The Seattle-area local survey included questions regarding prior HCV testing, diagnosis, and treatment. Both the national core NHBS and local supplement surveys were interviewer-administered. Interviewer-administered surveys were used to standardize question delivery and minimize variability in how items were presented, while also accommodating participants with literacy challenges.

Following survey completion, all participants were offered rapid HCV (OraQuick) and HIV antibody testing (Insti and Determine); all testing was voluntary. All participants were offered information on locations for additional HCV testing, care, and treatment, and educated on HCV transmission and reinfection. Participants who were HCV-positive were provided with options to link to care and treatment through an established primary care provider or anonymously referred to the Public Health - Seattle & King County HCV Program.

The NHBS and local surveys collected no personally identifiable information. All participants received $25 for completing the survey, $25 for completing HIV testing, $20 for completing HCV testing, and $20 for each enrollment referral (up to 5). The CDC and the Washington State Institutional Review Board (IRB) determined that NHBS is a surveillance activity and did not require further review or approval.

### Measures

We used data from the core survey to measure 2022 NHBS-PWID participant demographics including age (18–29, 30–39, 40–49, 50+), gender (female, male, or transgender), race/ethnicity (American Indian/Alaska Native, Asian, Black/African American, Hispanic/Latinx, Native Hawaiian or other Pacific Islander, White), housing status, health insurance status, and county of residence. Injection behavior and treatment in the past 12 months was also measured using data from the core and local surveys including primary drug injected (heroin, methamphetamine, heroin + methamphetamine, other), use of any local syringe services program, any syringe sharing, any non-injection drug use (excluding marijuana), receipt of medication for opioid use disorder, and health care visit. We also reported any alcohol use within the past 30 days. Finally, we calculated the proportion of participants who self-reported being HIV-positive and were ever told they had HCV. Among participants living with HIV, we estimated the proportion who reported currently being on antiretroviral therapy.

The primary outcomes for this study are self-reported steps along the HCV continuum of care including prior:

1. HCV testing (“Have you ever been tested for hepatitis C infection?”);
2. Diagnosis of HCV (“Has a doctor, nurse, or other health care provider ever told you that you had hepatitis C?”);
3. HCV treatment (“Have you ever taken medicine to treat your hepatitis C infection?”);
4. HCV cure (“Were you told you were cured of hepatitis C?”).

### Statistical Analysis

Prior to any analysis, the CDC and local survey team attempted to identify potential duplicate participants including looking for participants that had identical birthdays and other characteristics. If two participants had a high degree of matching, the second observation was removed. For the purposes of this study, the analytic sample was restricted to participants who had completed the core NHBS survey, the local survey supplement, and had valid rapid HCV antibody test results. Seeds were included in all analyses. For the HCV continuum of care, we calculated proportions and 95% confidence intervals (CI) to estimate the proportion of participants who had completed each step of the care continuum. First, we determined the proportion of participants who reported that they had received a prior HCV test and the proportion who had received a prior diagnosis of HCV among those who tested HCV antibody-positive. We then determined the proportion of participants who reported prior HCV treatment and HCV cure among those who had a previous diagnosis of HCV. Additionally, continuum of care data from the 2022 NHBS-PWID survey were compared to estimates from the 2018 Seattle-area NHBS-PWID survey using Pearson chi-square tests. While the results of the 2018 survey have previously been published [10], these analyses were re-run for this study for comparability.

Due to uncertainty in meeting assumptions required for RDS weights [15] — random recruitment from a participant’s social network and accurately estimating the size of their social network — we present results for unweighted data analysis. This is similar to the national NHBS-PWID report and prior publications [16].

## Results

### Sample and Characteristics

**Fig. 1:**
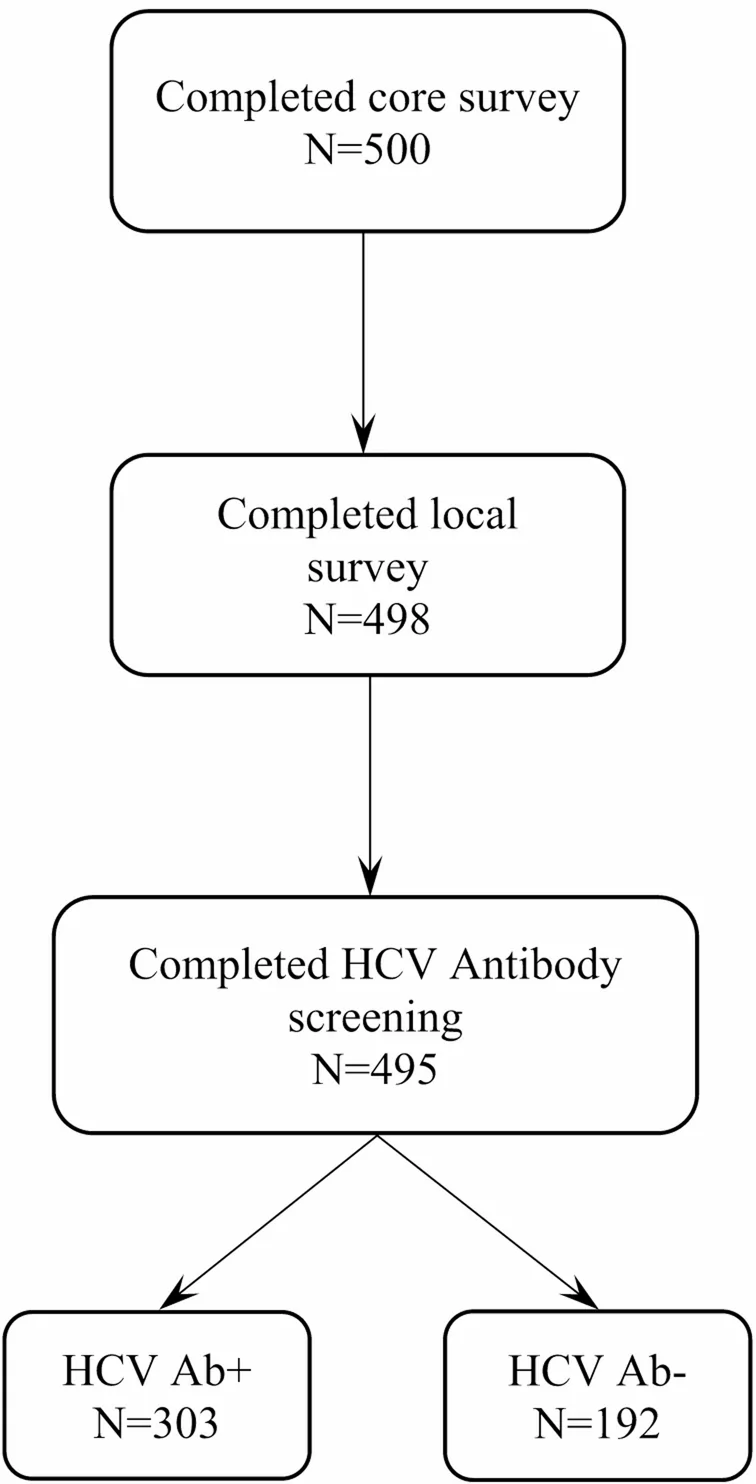
Seattle-area national HIV behavioral surveillance-people who inject drugs (NHBS-PWID) survey participants and hepatitis C virus (HCV) testing status, 2022

In 2022, a total of 500 PWID completed the core NHBS survey from King and Snohomish County, 22 of whom were RDS “seeds” (14 from King County, 8 from Snohomish County). Two participants did not complete the local survey, and three participants did not have valid HCV antibody results; these 5 participants were excluded from further analyses (Fig. 1). Of the 495 total participants who completed testing, 309 (62%) were male, 183 (37%) were female, and 3 (< 1%) were transgender (Table 1). The majority of participants (58%) were < 50 years of age. Participants could select more than one racial/ethnic identity, and 15% identified as American Indian/Alaska Native, 2% identified as Asian, 23% identified as Black/African American, 11% as Hispanic/Latinx, 7% as Native Hawaiian or other Pacific Islander, and 69% identified as White. Most participants were either currently homeless (45%) or had been homeless in the last 12 months (17%). Only 6% were currently uninsured. The majority (91%) lived in King County.

**Table 1:** Characteristics of Seattle-area National HIV Behavioral Surveillance-People Who Inject Drugs (NHBS-PWID) survey participants, 2022 (*N* = 495)

Characteristics		Total *N* = 495 *n* (%)
Demographics		
Age		
18–29		29 (5.9)
30–39		115 (23.2)
40–49		145 (29.3)
50+		206 (41.6)
Gender		
Male		309 (62.4)
Female		183 (37.0)
Transgender		3 (0.6)
Race/ethnicity^a^		
American Indian/Alaska Native		76 (15.5)
Asian		12 (2.4)
Black/African American		114 (23.2)
Hispanic/Latinx		53 (10.8)
Native Hawaiian or other Pacific Islander		32 (6.5)
White		338 (68.7)
Homeless		
No, not in the last 12 months		188 (38.0)
Yes, in the last 12 months		86 (17.4)
Yes, currently		221 (44.7)
Health insurance^a^		
Currently insured		465 (94.5)
Currently uninsured		27 (5.5)
County of residence		
King County		449 (90.7)
Snohomish County		46 (9.3)
Substance Use Behavior and Treatment		
Primary drug injected		
Heroin		209 (42.2)
Methamphetamine		140 (28.3)
Heroin + Methamphetamine		58 (11.7)
Other		88 (17.8)
Any local syringe services program use, past 12 months^b^		390 (79.0)
Any syringe sharing, past 12 months^b^		133 (26.9)
Any non-injection drug use, past 12 months		453 (91.5)
Any alcohol use, past 30 days		287 (58.0)
Medication for opioid use disorder, past 12 months^c^		291 (66.0)
Health care visit, past 12 months		400 (81.0)
HIV & HCV Testing Measures		
HIV+, self-reported		33 (6.7)
Current antiretroviral (ART) use, if HIV+		30 (90.9)
Ever told had HCV^d^		
No		199 (41.0)
Yes		227 (46.7)
Never tested		60 (12.4)

^a^3 people did not have data on race or health insurance

^b^1 person did not have data on local syringe services program use or syringe sharing

^c^Only asked to people who reported opioid use in the past 12 months

^d^9 people did not have data on self-reported HCV status

Heroin was the most commonly reported primary drug injected (42%) followed by methamphetamine (28%). In the past 12 months, most (79%) participants reported using any local syringe services program, 27% reported any syringe sharing, and 92% reported any non-injection drug use. Just over one-half (58%) reported any alcohol use in the past 30 days. Two-thirds of participants who used opioids (66%) reported medication for opioid use disorder (MOUD) in the past 12 months. The vast majority of participants (81%) reported any health care visit in the past 12 months. 7% reported having HIV; among those living with HIV, 91% reported current antiretroviral use. Approximately one-half (47%) had ever been told that they had HCV, 41% had been told they did not have HCV, and 12% had never been tested before. All participants consented to HCV testing. Of the 495 participants in our analytic sample, 303/495 (61%) were HCV antibody-positive.

### HCV Care Continuum

**Fig. 2:**
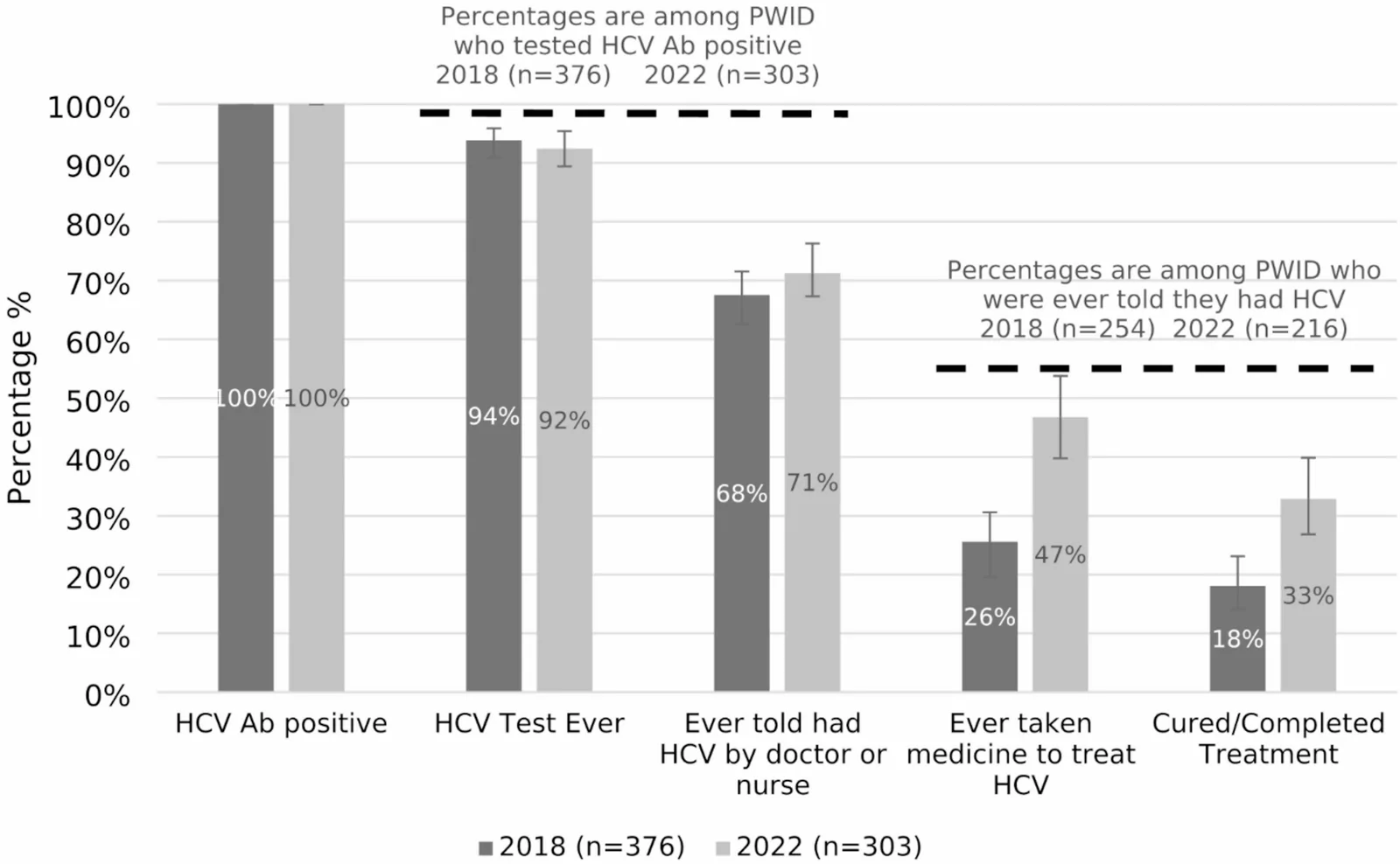
Hepatitis C virus (HCV) continuum of care among seattle-area national HIV behavioral surveillance-people who inject drugs (NHBS-PWID) survey participants, 2018 vs. 2022

Among the 303 participants who were HCV antibody-positive, 92% (280/303) reported any prior HCV test and 71% (216/303) reported a prior HCV diagnosis by a doctor or nurse (Fig. 2). Of those participants ever diagnosed with HCV, 47% (101/216) reported receiving treatment and 33% (71/216) reported being cured.

When comparing NHBS continuum of care data between 2018 and 2022, similar proportions of PWID reported a prior test for HCV [94% (95% CI: 91%−96%) vs. 92% (95% CI: 89%−95%), respectively, eχ^2^ = 0.5770, *p-value* = 0.447] and diagnosis of HCV [68% (95% CI: 63%−72%) vs. 71% (95% CI: 66%−76%), respectively, eχ^2^ = 1.0980, *p-value* = 0.295]. However, there was a significant difference in the proportion of PWID who had been told they had HCV who reported HCV treatment [26% (95% CI: 20%−31%) vs. 47% (95% CI: 40%−54%), eχ^2^ = 22.8977, *p-value* < 0.001] and cure [18% (95% CI: 14%−23%) vs. 33% (95% CI: 27%−40%), eχ^2^ = 13.6021, *p-value* < 0.001] when comparing data from 2018 to 2022, respectively.

## Discussion

This study includes an updated characterization of HCV infection status among PWID in the Seattle area. Our study showed that between 2018 and 2022, the proportion of PWID with HCV in the Seattle area who were treated and cured nearly doubled, providing evidence of progress towards HCV elimination. The large increase in the proportion of PWID who were treated and cured of HCV in the Seattle area signifies substantial headway towards Washington State’s HCV Elimination by 2030 goal. There are several policies that have been enacted to support HCV treatment for Washington State Medicaid beneficiaries, these include: allowing any licensed prescriber to screen and treat HCV in patients who have Medicaid, no prior authorization requirements for preferred DAA regimens, no drug abstinence or fibrosis requirements, and no need for labs six months apart to validate chronic infection [17]. These policies have been implemented in conjunction with an agreement between Washington State and AbbVie, who manufactures MAVYRET (glecaprevir/pibrentasvir), to provide glecaprevir/pibrentasvir to all Medicaid beneficiaries at no additional cost to the individuals [18]. Collectively, these policies have allowed for more expeditious treatment of patients and have paved the way for increased treatment uptake and HCV cure among PWID in the Seattle-area.

Our findings are in line with other studies that have also shown increases in treatment uptake among PWID globally. Valerio et al. reported that HCV treatment increased from 66% to 74% between 2018 and 2019 and 2019–2021, respectively, in the ETHOS Engage observational cohort study of PWID in Australia [19]. Among 2,436 PWID with chronic HCV infection in Norway, 1,118 (46%) had received HCV treatment between 2010 and 2019 (89% DAA-based) [20]. The U.S. is also seeing increases in cure rates of HCV. Among 399 participants with chronic HCV infection who self-reported HCV treatment in Baltimore, MD, HCV treatment uptake increased from 4% in 2014 to 59% in 2019 and 68% in 2020 [21]. Over this same period, the proportion with HCV viremia declined from 99% in 2014 to 44% in 2019 and 33% in 2020 [21].

Though we see these significant advances towards HCV treatment for all, Seattle remains short of the treatment goal set forth by WHO, which aims to treat 80% of those infected with HCV [7]. Substantial gaps remain in awareness and diagnosis of HCV among PWID. In our study, a meaningful proportion of participants who tested HCV antibody-positive had not previously been told they had HCV, highlighting persistent failures in diagnosis and communication of results. This finding is consistent with national estimates suggesting that approximately 40% of people living with HCV in the United States are unaware of their infection [22]. Prior studies among PWID have similarly documented gaps along the early stages of the HCV care continuum, with underdiagnosis driven by limited access to routine screening, fragmented care, and missed opportunities for testing in nontraditional settings such as syringe service programs and addiction treatment sites [7, 23]. Low awareness of HCV status represents a critical barrier to treatment initiation and undermines elimination efforts, as individuals who are undiagnosed cannot be linked to curative therapy [7]. Expanding routine and community-based HCV screening, coupled with prompt result notification and linkage to care, will be essential to closing these gaps and achieving HCV elimination targets.

Despite progress in HCV treatment and cure among PWID in the Seattle-area, multi-level barriers to treatment also still exist. According to Trooskin et al., the “silo effect” is a system-level barrier that occurs when there is either a lack of integration of services or inadequate sharing of information between care providers [24]. Regulation and reimbursement of certain treatments are often managed separately from one another, making it difficult to integrate care reimbursed through physical health payers with care reimbursed through behavioral health payers [24]. People living with HCV also experience disproportionate amounts of stigma which can lead to isolation and withdrawal from medical care [25]. Providers often have limited experience working with PWID and may have misperceptions about their ability to adhere to and complete treatment. Another barrier lies in the severe lack of trained specialists willing to treat HCV [25]. In a cross-sectional online survey of over 500 physicians, nurse practitioners, physician assistants, and clinical pharmacists who provide care to adult patients in Washington State conducted in 2022, lack of treating clinicians and clinician knowledge deficit were the most frequently identified barriers to treating HCV [13]. Barriers also exist at the patient-level including lack of education and awareness of HCV infection and/or treatment options, past discriminatory encounters with healthcare providers, and logistical/structural issues such as lack of transportation, housing instability, and incarceration, which can lead to missed appointments or loss to follow-up [24].

There are several limitations to this study. Data presented are from one, relatively high resourced, geographical area of the United States, and findings may not be generalizable to other settings. Similarly, the analysis was analyzed as a convenience sample and did not account for RDS recruitment, so estimates may be influenced by selection bias and may not fully represent the target population. Furthermore, data on prior HCV diagnosis, treatment, and cure were obtained through self-report and are subject to misclassification. The use of interviewer-administered surveys to collect information on sensitive topics such as sexual and drug-use behaviors may also suggest an underestimate as it can introduce social desirability bias, potentially leading to underreporting of stigmatized behaviors. Finally, this study does not include data from 2015 due to methodologic differences between the surveys and thus cannot provide information on longer time trends.

## Conclusions

Our study demonstrates substantial gains in treatment and cure of HCV among Seattle-area PWID between 2018 and 2022 yet highlights ongoing gaps that remain in the HCV continuum of care if Washington State is to meet HCV elimination goals by 2030. To achieve elimination of HCV by 2030, researchers, clinicians, and policymakers must continue to develop and implement novel HCV treatment interventions that aim to reduce barriers to HCV care and engage populations who have historically been marginalized and left out of traditional models of care.

## References

[1] World Health Organization. Hepatitis C [Internet]. WHO. 2024. https://www.who.int/news-room/fact-sheets/detail/hepatitis-c. Accessed 9 April 2024

[2] Mazumder H, Hossain MF, Shrestha P, Mahmud S, Husain M, et al. Prevalence and associated risk factors of current hepatitis C infection among U.S. general population and injection drug users aged 20–59 years: NHANES 2009–2018. PLoS One. 2024;19(8):e0309345. 39186570 10.1371/journal.pone.0309345 PMC11346729

[3] Hofmeister MG, Rosenthal EM, Barker LK, Rosenberg ES, Barranco MA, Hall EW, et al. Estimating Prevalence of Hepatitis C Virus Infection in the United States, 2013–2016. Hepatology. 2019;69(3):1020–31. 10.1002/hep.30297. Epub 2018 Nov 6. PMID: 30398671; PMCID: PMC6719781. 30398671 PMC6719781

[4] Falade-Nwulia O, Gicquelais RE, Astemborski J, McCormick SD, Kirk G, Sulkowski M, et al. Hepatitis C treatment uptake among people who inject drugs in the oral direct-acting antiviral era. Liver Int. 2020;40(10):2407–16. 10.1111/liv.14634. PMID: 32770638; PMCID: PMC7706292. 32770638 PMC7706292

[5] Hotton A, Mackesy-Amiti ME, Boodram B. Trends in homelessness and injection practices among young urban and suburban people who inject drugs: 1997–2017. Drug Alcohol Depend. 2021;225:108797. 10.1016/j.drugalcdep.2021.108797. 34102506 PMC9373853

[6] Mukhtar NA, Ness EM, Jhaveri M, Fix OK, Hart M, Dale C, et al. Epidemiologic features of a large hepatitis C cohort evaluated in a major health system in the western United States. Ann Hepatol. 2019;18(2):360–5. 10.1016/j.aohep.2018.12.003. 31053542

[7] Grebely J, Dore GJ, Morin S, Rockstroh JK, Klein MB. Elimination of HCV as a public health concern among people who inject drugs by 2030 - What will it take to get there? J Int AIDS Soc. 2017;20(1):22146. 10.7448/IAS.20.1.22146. 28782335 PMC5577699

[8] Basit H, Tyagi I, Koirala J, Hepatitis C, [Internet] SP. 2023. https://www.ncbi.nlm.nih.gov/books/NBK430897/. Accessed 6 March 2023

[9] Tsui JI, Miller CM, Scott JD, Corcorran MA, Dombrowski JC, Glick SN. Hepatitis C continuum of care and utilization of healthcare and harm reduction services among persons who inject drugs in Seattle. Drug Alcohol Depend. 2019;195:114–20. 10.1016/j.drugalcdep.2018.11.026. 30611979 PMC6440747

[10] Corcorran MA, Tsui JI, Scott JD, Dombrowski JC, Glick SN. Age and gender-specific hepatitis C continuum of care and predictors of direct acting antiviral treatment among persons who inject drugs in Seattle, Washington. Drug Alcohol Depend. 2021;220:108525. 10.1016/j.drugalcdep.2021.108525. 33461152 PMC7938869

[11] Tsui JI, Gojic AJ, Pierce KA, Tung EL, Connolly NC, Radick AC, et al. Pilot study of a community pharmacist led program to treat hepatitis C virus among people who inject drugs. Drug Alcohol Depend Rep. 2023;10:100213. PMID: 38261893; PMCID: PMC10796962. 38261893 10.1016/j.dadr.2023.100213 PMC10796962

[12] Cooper MP, Foley H, Damico D, Wright M, Rhudy C, Schadler A, et al. Impact of the COVID-19 pandemic on hepatitis C outcomes at a health-system specialty pharmacy. J Manag Care Spec Pharm. 2022;28(6):667–72. PMID: 35621721; PMCID: PMC10372976. 35621721 10.18553/jmcp.2022.28.6.667 PMC10372976

[13] Cox-North P, Wiggins L, Stockton J, Huriaux E, Fliss M, Evaskus L, et al. Provider reported implementation barriers to hepatitis C elimination in Washington State. BMC Prim Care. 2024;25(1):252. 10.1186/s12875-024-02507-0. PMID: 38992590; PMCID: PMC11241921. 38992590 PMC11241921

[14] Kanny D, Broz D, Finlayson T, Lee K, Sionean C, Wejnert C, et al. A key comprehensive system for biobehavioral surveillance of populations disproportionately affected by HIV (National HIV Behavioral Surveillance): cross-sectional survey study. JMIR Public Health Surveill. 2022;8(11):e39053. 10.2196/39053. 36378503 PMC9709677

[15] Li J, Valente TW, Shin HS, Weeks M, Zelenev A, Moothi G, et al. Overlooked Threats to Respondent Driven Sampling Estimators: Peer Recruitment Reality, Degree Measures, and Random Selection Assumption. AIDS Behav. 2018;22(7):2340–59. 10.1007/s10461-017-1827-1. PMID: 28660381; PMCID: PMC5745307. 28660381 PMC5745307

[16] Centers for Disease Control and Prevention. HIV infection risk, prevention, and testing behaviors among persons who inject drugs—National HIV behavioral surveillance, 20, Cities US. 2022. HIV Surveillance Special Report 35. https://www.cdc.gov/hiv/library/reports/hiv-surveillance.html. Published February 2024. Accessed Feb 2026.

[17] Washington State Department of Health. Hep C Free Washington Urgent: Test and treat people who use drugs for hepatitis C [Internet]. Washington State Healthcare Authority; February 2021. DOH 150 – 146. https://doh.wa.gov/sites/default/files/legacy/Documents/Pubs//150-146-HepC-cliniciantestingbrief.pdf. Accessed Feb 2021.

[18] Washington State Healthcare Authority. Washington State Health Care Authority and partners reach milestone toward eliminating hepatitis C [Internet]. 2024 May 15. https://www.hca.wa.gov/about-hca/news/news-release/washington-state-health-care-authority-and-partners-reach-milestone-toward-eliminating-hepatitis-c#:~:text=In%202019%2C%20HCA%20contracted%20with,Apple%20Health%20(Medicaid)%20clients. Accessed 15 May 2024.

[19] Valerio H, Alavi M, Conway A, Silk D, Treloar C, Martinello M, et al. Declining prevalence of current HCV infection and increased treatment uptake among people who inject drugs: The ETHOS Engage study. Int J Drug Policy. 2022;105:103706. Epub 2022 May 6. PMID: 35533635. 35533635 10.1016/j.drugpo.2022.103706

[20] Malme KB, Ulstein K, Finbråten AK, Wüsthoff LEC, Kielland KB, Hauge J, et al. Hepatitis C treatment uptake among people who inject drugs in Oslo, Norway: A registry-based study. Int J Drug Policy. 2023;116:104044. Epub 2023 May 5. PMID: 37149914. 37149914 10.1016/j.drugpo.2023.104044

[21] Coyle CR, Gicquelais RE, Genberg BL, Astemborski J, Falade-Nwulia O, Kirk GD, et al. Temporal trends in HCV treatment uptake and success among people who inject drugs in Baltimore, MD since the introduction of direct acting antivirals. Drug Alcohol Depend. 2023;253:111007. Epub 2023 Oct 21. PMID: 38456165; PMCID: PMC10917145. 38456165 10.1016/j.drugalcdep.2023.111007 PMC10917145

[22] Gnanapandithan K, Ghali MP. Self-awareness of hepatitis C infection in the United States: a cross-sectional study based on the National Health Nutrition and Examination Survey. PLoS One. 2023;18(10):e0293315. 10.1371/journal.pone.0293315. 37874815 PMC10597475

[23] Balsom CR, Farrell A, Kelly DV. Barriers and enablers to testing for hepatitis C virus infection in people who inject drugs - a scoping review of the qualitative evidence. BMC Public Health. 2023;23(1):1038. 10.1186/s12889-023-16017-8. 37259073 PMC10234098

[24] Trooskin SB, Dore G, Kostman J. We Must Do Better: Addressing HCV Treatment Barriers in Persons Who Inject Drugs in the United States. J Infect Dis. 2020;222(Suppl 9):S773–81. 10.1093/infdis/jiaa574. 33245349

[25] Washington State Department of Health. Hep C Free Washington: Plan to Eliminate Hepatitis C in Washington State by 2030 [Internet]. Division of disease control & health statistics. 2019 July. 150-nonDOH. https://doh.wa.gov/sites/default/files/legacy/Documents/Pubs/150nonDOH-HepCFreeWA-PlanJuly2019.pdf. Accessed July 2019.

